# Insights from a computational analysis of the SARS‐CoV‐2 Omicron variant: Host–pathogen interaction, pathogenicity, and possible drug therapeutics

**DOI:** 10.1002/iid3.639

**Published:** 2022-06-21

**Authors:** Md Sorwer Alam Parvez, Manash Kumar Saha, Md. Ibrahim, Yusha Araf, Md. Taufiqul Islam, Gen Ohtsuki, Mohammad Jakir Hosen

**Affiliations:** ^1^ Department of Drug Discovery Medicine Kyoto University Graduate School of Medicine Kyoto Japan; ^2^ Department of Genetic Engineering & Biotechnology Shahjalal University of Science & Technology Sylhet Bangladesh

**Keywords:** ACE2, COVID‐19, drugs efficacy, host–pathogen interaction, NRP1, Omicron variant

## Abstract

**Introduction:**

Prominently accountable for the upsurge of COVID‐19 cases as the world attempts to recover from the previous two waves, Omicron has further threatened the conventional therapeutic approaches. The lack of extensive research regarding Omicron has raised the need to establish correlations to understand this variant by structural comparisons. Here, we evaluate, correlate, and compare its genomic sequences through an immunoinformatic approach to understand its epidemiological characteristics and responses to existing drugs.

**Methods:**

We reconstructed the phylogenetic tree and compared the mutational spectrum. We analyzed the mutations that occurred in the Omicron variant and correlated how these mutations affect infectivity and pathogenicity. Then, we studied how mutations in the receptor‐binding domain affect its interaction with host factors through molecular docking. Finally, we evaluated the drug efficacy against the main protease of the Omicron through molecular docking and validated the docking results with molecular dynamics simulation.

**Results:**

Phylogenetic and mutational analysis revealed the Omicron variant is similar to the highly infectious B.1.620 variant, while mutations within the prominent proteins are hypothesized to alter its pathogenicity. Moreover, docking evaluations revealed significant differences in binding affinity with human receptors, angiotensin‐converting enzyme 2 and NRP1. Surprisingly, most of the tested drugs were proven to be effective. Nirmatrelvir, 13b, and Lopinavir displayed increased effectiveness against Omicron.

**Conclusion:**

Omicron variant may be originated from the highly infectious B.1.620 variant, while it was less pathogenic due to the mutations in the prominent proteins. Nirmatrelvir, 13b, and Lopinavir would be the most effective, compared to other promising drugs that were proven effective.

## Graphical abstract

Parvez et al. investigate genomic sequences and drug efficacy via a bioinformatic approach with the SARS‐CoV‐2 Omicron variant. At first, they perform a comparative analysis of Omicron variant with other variants, and then they evaluate the binding affinity with human receptors, ACE2, NRP1, BSG and DPP4. Finally, they analyze the drug effectiveness against the Omicron targeting Main Protease, and found that Nirmatrelvir (Paxlovid), 13b, and Lopinavir displayed increased effectiveness and efficiency

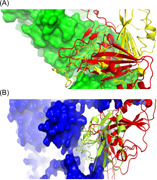

## 1 INTRODUCTION

COVID‐19 pandemic by the SARS‐CoV‐2 (also known as coronavirus) has pulverized the health care system of the world since November 2019, which changed our lives and caused strict measures to prevent the spread of infection.^1^ Currently, most countries are threatened by the third to sixth wave of this severe acute respiratory disease, and the entire world is trying to combat it.^2^ It is a situation constantly changing and evolving.^3^ Multiple forms of this virus, including alpha, beta, gamma, and delta have shown their rampage, and most recently, the Omicron form is circulating over the world with a hot spot of more than 30 mutations in the spike protein.^4^, ^5^

Omicron, a newly evolved and very highly infectious coronavirus variant (B.1.1.529), was designated as a variant of serious concern by the World Health Organization on November 26, 2021.^6^ Since the first case report in Botswana on November 11, 2021, Omicron has spread to 108 countries and infected 150,000 patients within a month, despite greater surveillance. While it is too early to assess exact severity, preliminary findings suggest that Omicron has a less clinical presentation and 4.9% lower hospital admission rates.^7^ It is the most highly altered version, similar to those reported in earlier variants of concern, linked to its increased transmissibility and partial resistance to vaccine‐induced immunity.^6^, ^8^, ^9^ Omicron was born into a COVID‐19‐weary world and repleted with further anxiety and distrust at the pandemic's extensive detrimental social, emotional, and economic consequences.^10^

The laboratories chasing the Omicron variant have yet thoroughly defined its epidemiologic characteristics. The features of DNA sequence alone cannot be used to determine them, which causes a diagnostic challenge. Concerning the spike protein of the Omicron variant, mutations were reported in the S protein. The alterations in the S protein receptor‐binding domain (RBD) may influence its infectivity and antibody resistance, as RBD is necessary for binding with host angiotensin‐converting enzyme 2 (ACE2) during the early infection process. The binding free energy (BFE) between the S protein RBD and the ACE2 has been demonstrated proportional to viral infectivity in several investigations. Moreover, mutations in the nucleocapsid protein have also been reported in Omicron, which helps viral proliferation.^9^ Increased transmissibility, better viral binding affinity, and higher antibody escape would have all been linked to these mutations.^4^

The Omicron variant has currently become a great concern for the world. Basic research is required to unveil its molecular consequences via gene mutations, which resulted in changes in infectivity, pathogenicity, and antigenic escape potential.^11^ Both occurrence area and the variant of Omicron origin are also unclear. To face the challenge of Omicron, it is urgent to test possible therapeutics and the effectiveness of available vaccines. Scientific data are required to have substantial benefits to advance the medical practice.^12^ Therefore, our study aimed to elucidate the novelty of Omicron from other variants: molecular mechanism of its high infectious ability and less pathogenicity. We also analyzed the effectiveness of current promising drugs against this variant. Our findings should provide novel insights on the structural and functional impact of mutations in Omicron, the impact during host interaction, and possible therapeutics for combatting this highly infectious variant.

## 2 MATERIALS AND METHODS

### 2.1 Retrieval of the sequences

The complete genome sequences of all notable SARS‐CoV‐2 variants, including the variant of concern and the variant of interest, were retrieved from the GISAID database (www.gisaid.org). We collected a total number of 30 variants along with South Africa variant B.1.1.529 (Omicron) from this database. Additionally, the sequence of the Wuhan SARS‐CoV‐2 was also retrieved and considered as the reference for the comparative analysis.

### 2.2 Multiple sequence alignment (MSA) and phylogenetic tree reconstruction

We performed MSA using MUSCLE v.5 alignment tools.^13^ Further, we used this MSA file for reconstructing a phylogenetic tree. IQ‐TREE v.2 was adopted for the reconstruction of the tree with maximum‐likelihood (ML) method.^14^ To identify the best‐fit substitution model, we used ModelFinder for the model test (279 models) and selected the best‐fit substitution model (GTR+F+R2) based on Bayesian Information Criterion (BIC), which is a criterion for model selection, and a model with lower BIC is generally considered as a good model.^15^ BIC is calculated with the following equations:

BIC=kln(N)−2ln(L)

where *k*, number of parameters estimated by the model; *N*, the number of data points in the number of observations or equivalent to the sample size; *L*, the maximized value of the likelihood function of the model.

Besides, we performed both shimodaira‐hasegawa like approximate likelihood ratio test and ultrafast bootstrap to assess branch supports where both were set to 1000. UFBoot2 was used for this bootstraps assessment operation.^16^ Finally, we employed an iTOL v.6 online tool for the visualization and analysis of the reconstructed phylogenetic tree.^17^

### 2.3 Identification of the nucleotide variations

The MSA file was analyzed by MEGAX software to identify the nucleotide variations in all variants, considering the Wuhan strain as a reference.^18^

### 2.4 Prediction of the encoded proteins and identification of the variations

To predict the genes and their encoded proteins in the variant genome, we used FGENESV (uses pattern recognition and Markov chain models) of Softberry (http://linux1.softberry.com/berry.phtml) viral gene prediction tools. The predicted genes and proteins were further confirmed using the Basic Local Alignment Search Tool (BLAST) of NCBI (https://blast.ncbi.nlm.nih.gov/Blast.cgi). By adopting Clustal omega, we further performed a pairwise alignment of each protein with its corresponding protein of the reference strain to identify the amino acid variations, and we visualized and analyzed it using MVIEW.^19^, ^20^

### 2.5 Modeling of the mutant RBD of spike protein and validation

The 3D crystal structure of wild RBD of spike protein was retrieved from the Protein Data Bank database (https://www.rcsb.org/) with the accession number of PDB ID: 6M17.^21^ This structure was cleaned by removing water, ligand, and other complexed molecules using the PyMOL.^22^ We modeled the 3D structure of mutant RBD of Omicron variant using SWISS‐MODEL (https://swissmodel.expasy.org/) webserver.^23^ Then, the generated 3D structure was validated by using ERRAT and PROCHECK with the SAVES v6.0 server (https://saves.mbi.ucla.edu/).^24^, ^25^

### 2.6 Molecular docking of RBD of spike protein with human receptors

We analyzed the interaction of both wild and mutant RBD of spike protein with human receptors ACE2, NRP‐1, BSG, and DPP4 through protein–protein molecular docking using FRODOCK tools.^26^ We selected these four human receptors, as previous studies demonstrated that spike protein interacts with them. The 3D structure of the receptor proteins was retrieved from the PDB except for NRP‐1. Due to the unavailability of the high coverage NRP‐1 3D structure in the PDB, we retrieved it from the Alphafold database.^27^ Then, we prepared the input PDB file by converting it to PQR format using PDB2PQR, via a Python‐based structural conversion utility.^28^ We used CHARMM force field during the protein–protein docking simulation.^29^ Finally, we calculated and obtained the binding energy of the binding interaction by using the PDBe PISA v.1.52 server (https://www.ebi.ac.uk/msd-srv/prot_int/cgi-bin/piserver).

### 2.7 Analysis of the effectiveness of promising drugs

We analyzed 10 promising drugs targeting the main (3CL) protease protein of SARS‐CoV‐2 including Nirmatrelvir, Ritonavir, Ivermectin, Lopinavir, Boceprevir, 13b, N3, GC‐373, GC376, and PF‐00835231.^30^, ^31^, ^32^ The PDB structures of these drugs were retrieved from the DrugBank (https://go.drugbank.com/) and PubChem (https://pubchem.ncbi.nlm.nih.gov/).^33^, ^34^ The 3D structure of the wild main protease was retrieved from PDB ID: 6WTK. We modeled the mutant by SWISS‐MODEL.^23^ Before the molecular docking, we removed water molecules, ligands, and other complex molecules from the 3D structures. Polar hydrogen atoms and required charges for the energy minimization were further added. Molecular docking was performed by using AutoDock Vina tools.^35^ We set the parameters of the grid box to size 40 Å × 64 Å × 64 Å (*x*, *y*, and *z*) and center −16.773 × −15.229 × 13.709 (*x*, *y*, and *z*) with the spacing 1. The exhaustiveness was set to 8. Further, we used PyMOL for the analysis and visualization of the protein–ligand complex molecules.^22^ The 2D diagrams of protein–ligand interaction were generated with Discovery Studio.^36^

### 2.8 Molecular dynamics simulation (MDS)

**Figure 1:**
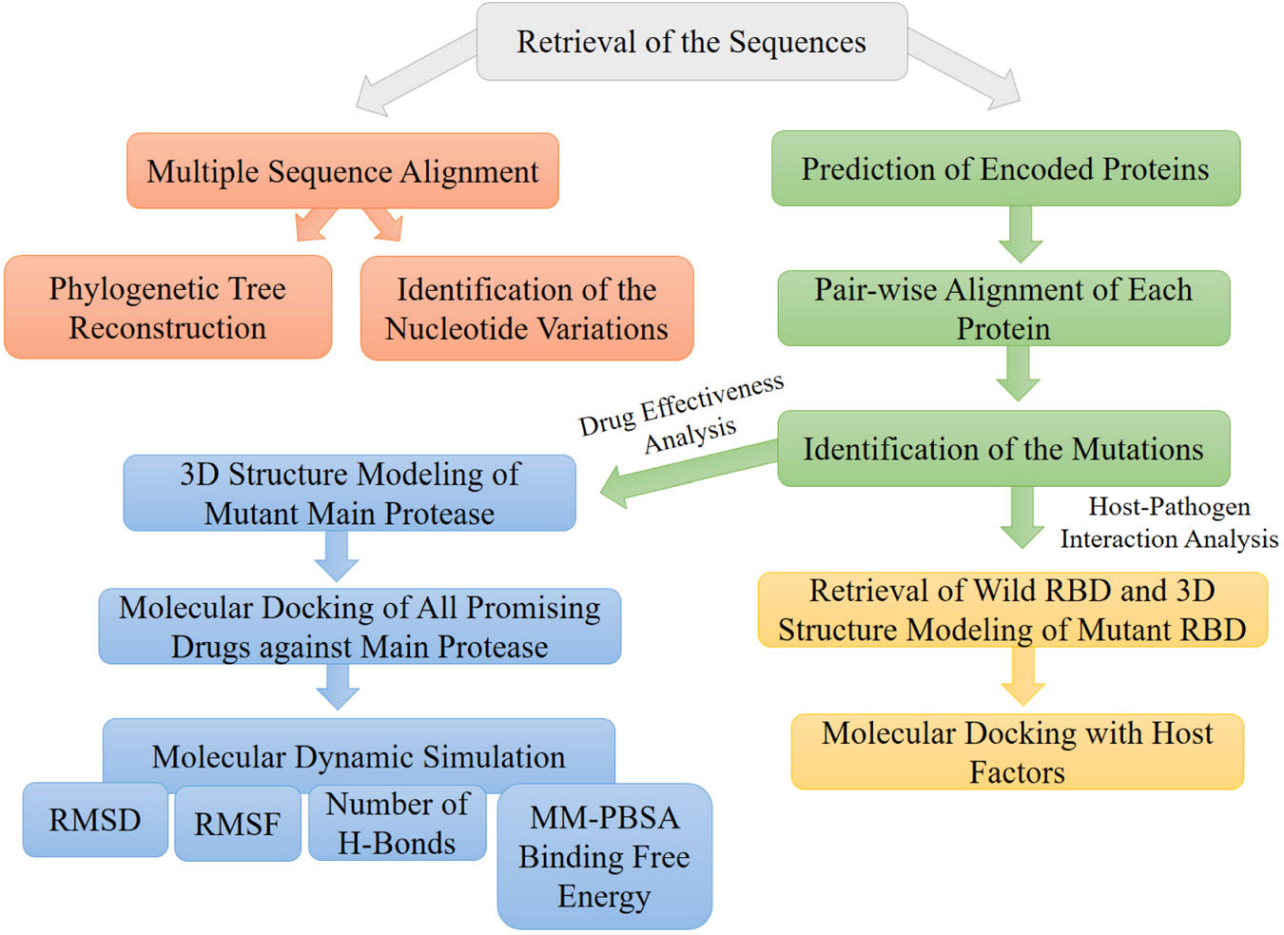
The schematic representation of the complete workflow. First, all sequences were retrieved, and multiple sequence alignment were performed. Then, phylogenetic tree were reconstructed, and nucleotide variations were identified. Further, all the encoded proteins were predicted followed by identifying of the mutations in each protein through pair‐wise alignment with the reference protein. Last, host–pathogens interaction and drug effectiveness analysis were done. For the host–pathogen analysis, the mutant RBD of spike Protein of Omicron was modeled and docked against host factors. Drug effectiveness was analyzed against a potent drug targets main protease by 3D structure modeling of the protein followed by molecular docking and molecular dynamics simulation analysis. MM‐PBSA, Molecular Mechanic/Poisson–Boltzmann Surface Area; RBD, receptor‐binding domain; RMSD, root mean square deviation; RMSF, root mean square fluctuation.

MDS was done, by employing GROMACS 2018 to analyze and validate the docked poses of the top drugs complexed with the main protease.^37^ The time interval for this simulation was 100 ns. GROMOS96 43a force field and TIP4P water model were used for this simulation while NaCl salt was used for the neutralization.^38^, ^39^ We used the steepest descent method with 5000 steps in all minimization processes and set the temperature to 300 K. The approximate number of frames per simulation was 1000. Root mean square deviation (RMSD), root mean square fluctuation (RMSF), and numbers of hydrogen bonds (H‐bonds) were computed over the simulation time. Last, we calculated the BFE with the Molecular Mechanic/Poisson–Boltzmann Surface Area (MM‐PBSA) approach utilizing the script “g_MMPBSA” of GROMACS.^40^ The potential energy of solvation, including electrostatic (∆*E*_elec_) and van der Waals energy (∆*E*_vdw_), and the total BFE (∆*G*_bind_), was determined to understand the binding energy of the interaction between the drugs and the receptor. We summarized the complete workflow in Figure 1.

## 3 RESULTS

### 3.1 Phylogenetic analysis

**Figure 2:**
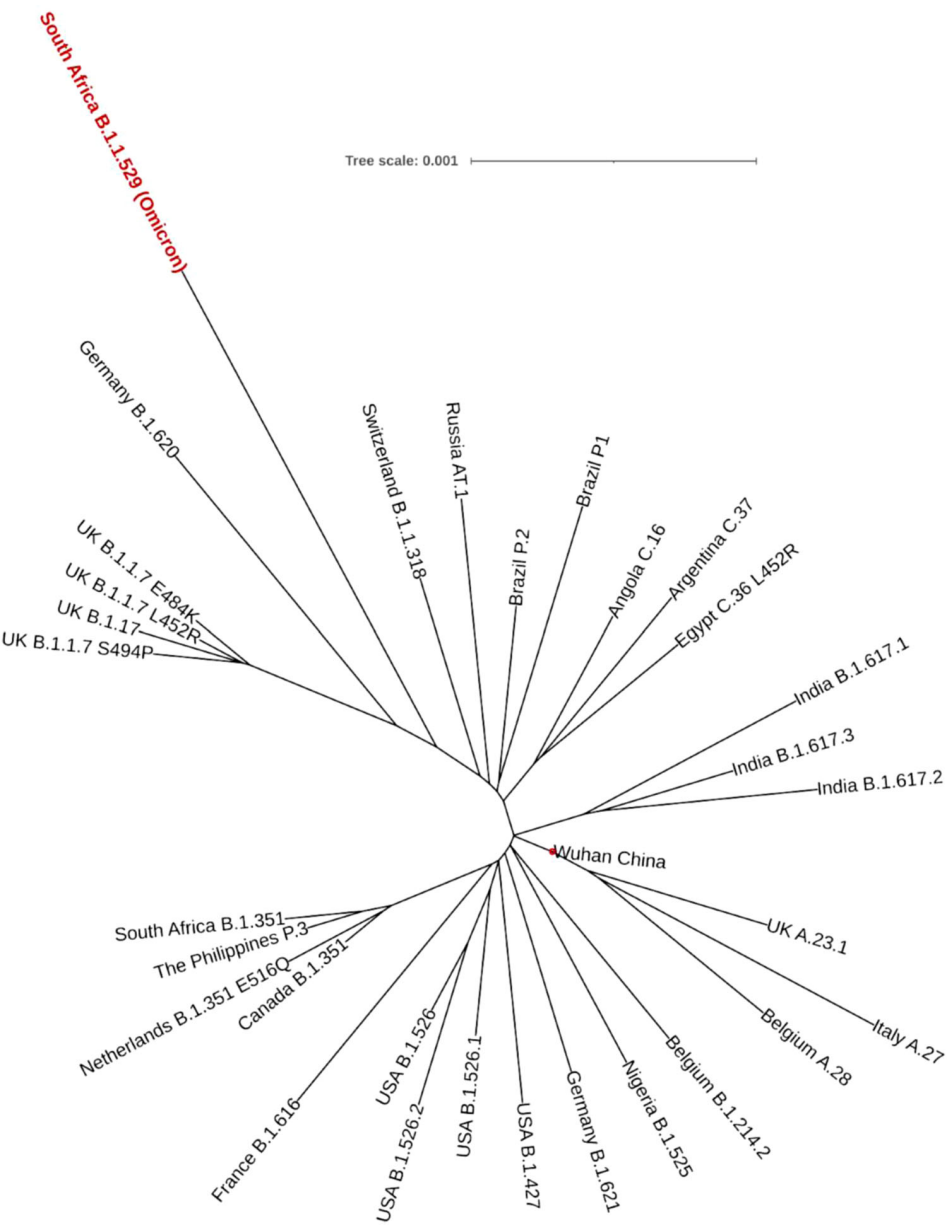
Maximum‐likelihood unrooted phylogenetic tree of all notable SARS‐CoV‐2 variants. South Africa B.1.1.529 (Omicron) was very close to Germany B.1.620. All the variants were centered with the Wuhan strain (red circle). A scale indicates genetic variation, defined as the number of substitutions per nucleotide site, here 0.001 substitutions per nucleotide position.

First, we reconstructed a phylogenetic tree from the MSA of all notable variants, including South Africa B.1.1.529 (Omicron). We further reconstructed an unrooted phylogenetic tree through ML methods to find closely related variants. Surprisingly, we found that the Omicron variant was very closely related to SARS‐CoV‐2 variants of Germany B.1.620 (Figure 2). Interestingly, the Switzerland B.1.1.318 variant was also localized close to the Omicron variant. All variants were centered at the Wuhan strain (Figure 2, red circle) that was the very early strain of SARS‐CoV‐2.

### 3.2 Identification of the nucleotide variations

Variations in the genome sequences of all notable variants from the alignment file were identified by comparing them with the Wuhan strain. We found that the variant South Africa B.1.1.529 (Omicron), UK B.1.1.7+S494P, and Russia AT.1 were highly mutated (Table 1). However, most of the mutations in the UK B.1.1.7+S494P and Russia AT.1 variant were synonymous, and Omicron contained the maximum number of nonsynonymous mutations (50 nonsynonymous mutations). Remarkably, we found most of these nonsynonymous mutations to locate in the spike protein sequence. Notably, some of the deletions and insertions were found in the consecutive bases of Omicron, which affect encoded proteins (Table 2).

**Table 1:** Nucleotide variations in all notable variants.

Variants name	Type	Total mutations	Insertions	Deletions	Nonsynonymous
South Africa B.1.1.529	Omicron	117	9	52	50
UK B.1.17	Alpha	58	0	19	23
UK B.1.1.7+E484K	Alpha	55	0	19	20
UK B.1.1.7+L452R	Alpha	52	0	19	20
UK B.1.1.7+S494P	Alpha	210	0	173	23
South Africa B.1.351	Beta	48	0	18	11
Netherlands B.1.351+E516Q	Beta	59	0	28	23
Canada B.1.351	Beta	42	0	18	20
India B.1.617.2	Delta	35	0	0	22
USA B.1.427	Epsilon	32	0	0	21
Nigeria B.1.525	Eta	54	0	24	20
Brazil P1	Gamma	38	0	0	12
USA B.1.526	Iota	32	0	10	15
USA B.1.526.1	Iota	38	0	13	21
USA B.1.526.2	Iota	77	0	48	6
India B.1.617.1	Kappa	36	0	0	24
India B.1.617.3	Kappa	27	0	0	22
Germany B.1.621	Mu	29	0	0	35
France B.1.616	Other	69	0	32	25
The Philippines P.3	Other	46	0	18	18
Egypt C.36+L452R	Other	37	0	6	25
Russia AT.1	Other	144	12	95	15
Switzerland B.1.1.318	Other	61	0	30	24
UK A.23.1	Other	23	0	0	15
Angola C.16	Other	29	0	0	18
Belgium A.28	Other	33	0	6	16
Italy A.27	Other	55	12	6	19
Germany B.1.620	Other	67	0	18	15
Argentina C.37	Other	42	0	9	14
Belgium B.1.214.2	Other	69	9	32	19
Brazil P.2	Zeta	46	0	19	13

**Table 2:** Consecutive deletions and insertions in Omicron variant.

Mutation types	Consecutive base	Position	Effected protein
Deletions	3	6513–6515	Papain like protease Nsp3
Deletions	9	11288–11296	Nsp6
Deletions	6	21765–21770	Spike
Deletions	9	21987–21995	Spike
Deletions	3	22194–22196	Spike
Insertion	9	22205–22213	Spike
Deletions	9	28395–28403	Nucleocapsid phosphoprotein

Abbreviations: Nsp3, nonstructural protein 3; Nsp6, nonstructural protein 6.

### 3.3 Identification of the mutations in the proteins

FGENESV and further pairwise alignment analyses indicated that the Omicron variant had mutations across polyprotein ab, spike protein, envelope protein, membrane glycoprotein, and nucleocapsid phosphoprotein (Table 3). Most numbers of the mutations were found located in spike protein. In the case of polyprotein ab, mutations occurred in papain‐like protease nsp3, nsp4, 3C‐like protease nsp5, nsp6, RNA dependent RNA polymerase nsp12, and proofreading exoribonuclease nsp14. Interestingly, deletions of three consecutive amino acids at positions 31–33 were detected in nucleocapsid phosphoprotein.

**Table 3:** Mutations in the proteins encoded by Omicron variants.

Protein name	Mutations
Polyprotein ab (papain‐like protease Nsp3)	Del:S2083; L2084I; A2710T
Polyprotein ab (Nsp4)	T3255I
Polyprotein ab (3C‐like protease Nsp5)	P3395H
Polyprotein ab (Nsp6)	Del:L3674; Del:S3675; Del;G3676; I3758V
Polyprotein ab (RNA‐dependent RNA polymerase Nsp12)	P4715L
Polyprotein ab (proofreading exoribonuclease nsp14)	I5967V
Spike	A67V; Del:H69; Del:V70; T95I; G142D; Del:V143; Del:Y144; N211I; L212V; In:213RE; V213P; R214E; G339D; S371L; S373P; S375F; K417N; G445S; S477N; T478K; E484A; Q493R; G496S; Q498R; N501Y; Y505H; T547K; D614G; H655Y; N679K; P681H; D796Y; N856K; Q954H; N969K; L981F
Envelope protein	T9I
Membrane glycoprotein	D3G; Q19E; A63T
Nucleocapsid phosphoprotein	P13L; Del:E31; Del:R32; Del:S33; R203K; G204R

### 3.4 Modeling of the mutant RBD of spike protein and validation

**Figure 3:**
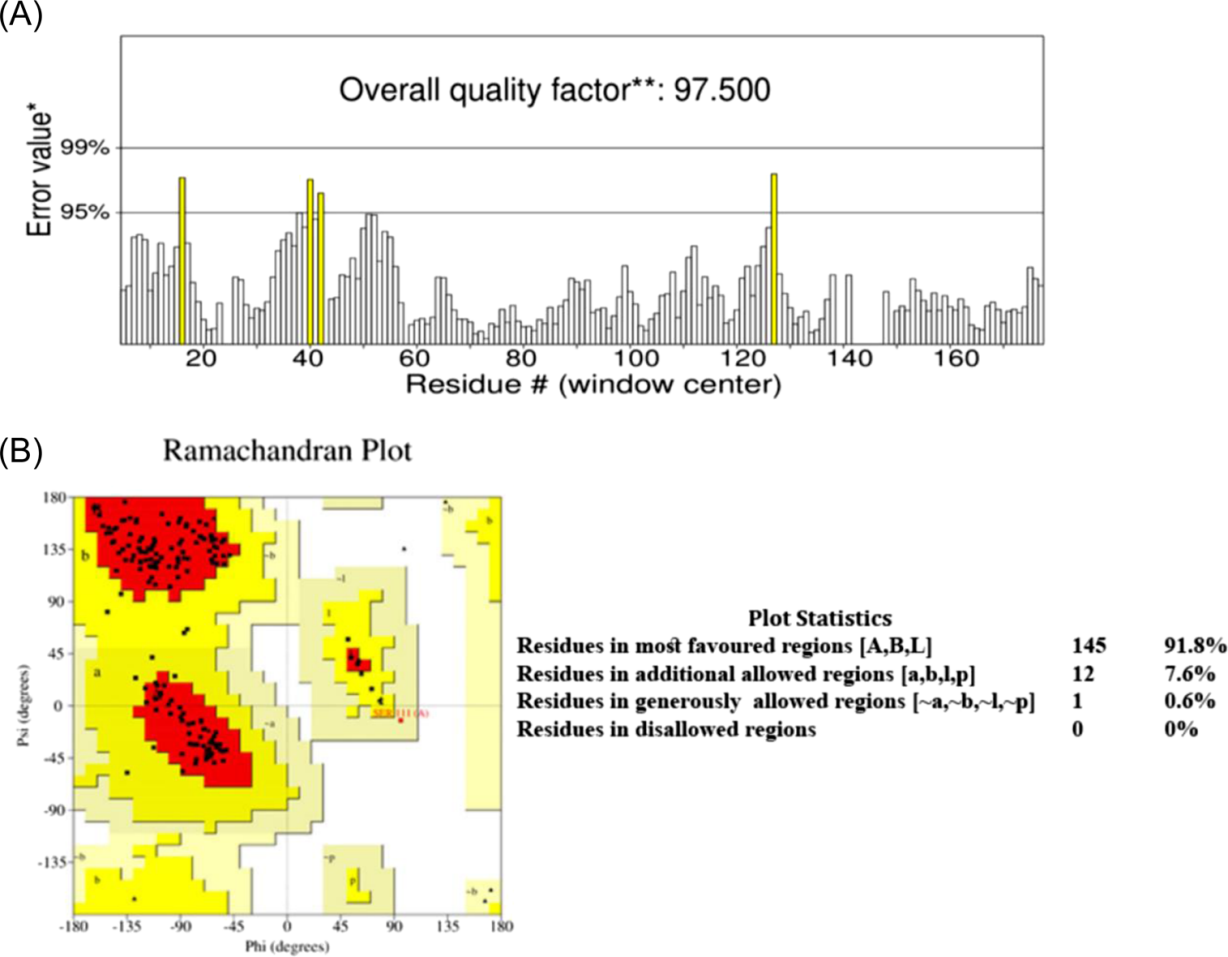
Validation of the 3D model. (A) ERRAT validation assessment. The overall quality factor was 97.5. (B) Ramachandran Plot by PROCHECK. One hundred and forty‐five residues (91.8%) were found in most favored regions while 12 residues (7.6%) were in additional allowed regions of the plot. No residues were found in disallowed regions.

The 3D modeling of mutant RBD of spike protein was done by SWISS‐MODEL homology modeling using wild‐type RBD of spike protein (with PDB ID 6M17) as a template. We further validated the obtained 3D model through ERRAT and PROCHECK tools. ERRAT validation revealed the overall quality factor of this model was 97.5 (Figure 3). Ramachandran plot analysis by PROCHECK revealed that 91.8% of its residues were in the most favored regions, and 7.6% were in additional allowed regions (Figure 3). A model would be considered as good quality and high‐reliability if it has over 90% of its residues in the most favored regions. All these validation scores suggested that this model was highly reliable to use for further analysis.

### 3.5 Molecular docking of RBD of spike protein with human receptors

**Figure 4:**
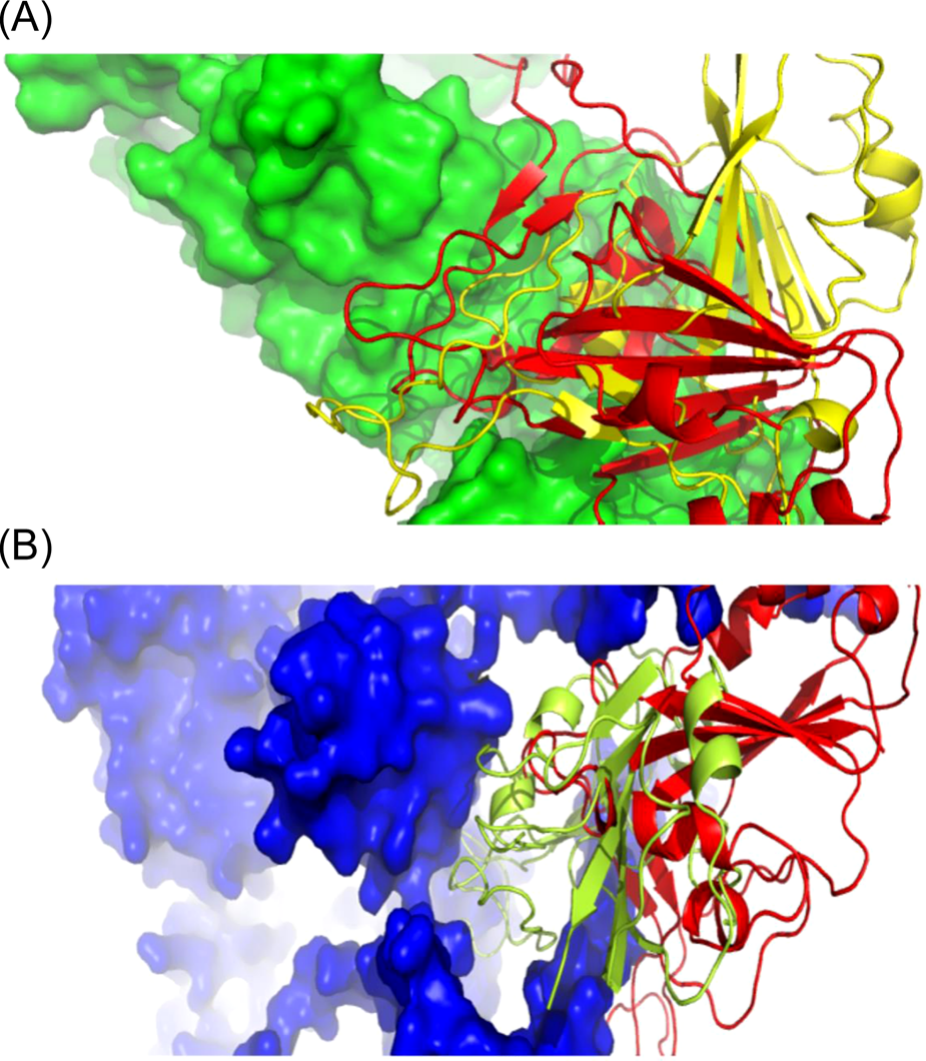
Interaction of wild‐type and mutant RBD with human receptors. (A) Interaction of RBD with ACE2. Here, green color represents ACE2, yellow color for wild‐type RBD, and red color for mutant RBD. (B) Interaction of RBD with NRP1. Here, blue color represents NRP1, yellow color for wild‐type RBD, and red color for mutant RBD. ACE2, angiotensin‐converting enzyme 2; RBD, receptor‐binding domain.

Next, we performed molecular docking analysis to investigate the impact of mutations in the RBD spike protein in interaction with human receptors. We used four previously reported receptors, including ACE2, NRP‐1, DPP4, and BSG, in this analysis.^41^, ^42^, ^43^, ^44^ Docking analysis revealed that the binding energy for interaction with ACE2 was decreased from −15.9 to −17.2 while increased for NRP1 from −27.2 to −22.9, compared to wild‐type RBD (Table 4 and Figure 4). The other two receptors showed higher binding energy in both wild‐ and mutant‐type RBD, compared to ACE2 and NRP1.

**Table 4:** Binding energy of interaction between wild and mutant RBD with human receptors.

Human receptors	Wild (kcal/mol)	Omicron (kcal/mol)
ACE2	−15.9	−17.2
NRP‐1	−27.2	−22.9
DPP4	−12.4	−12.3
BSG	−6.1	−8.5

Abbreviations: ACE2, angiotensin‐converting enzyme 2; RBD, receptor‐binding domain.

### 3.6 Effectiveness of the promising drugs

**Figure 5:**
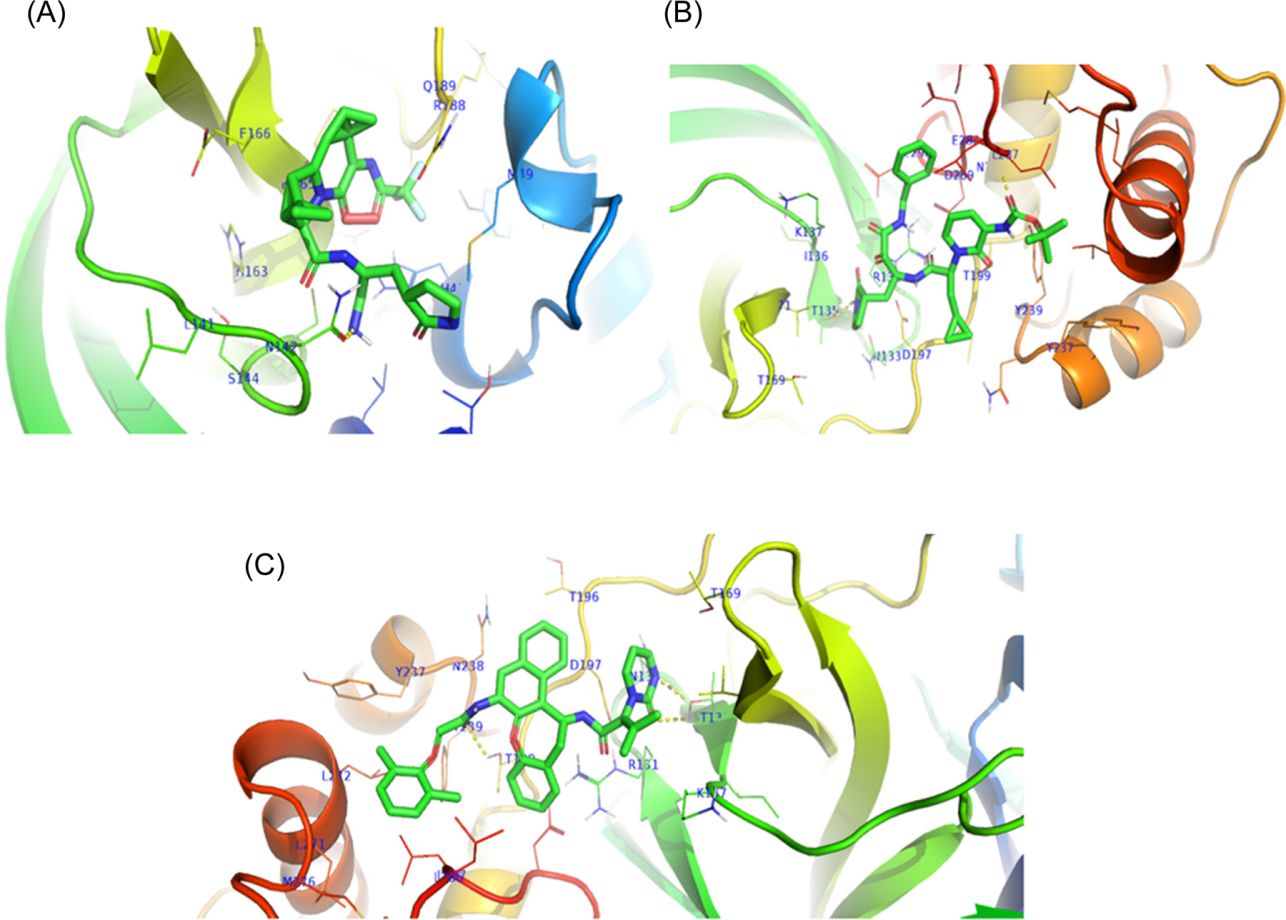
Interaction of drugs with mutant main protease of Omicron variant. Amino acids of the binding site was presented with blue color. Here, (A) interaction of Nirmatrelvir, (B) interaction of 13b, and (C) interaction of Lopinavir with main protease of Omicron variants.

**Figure 6:**
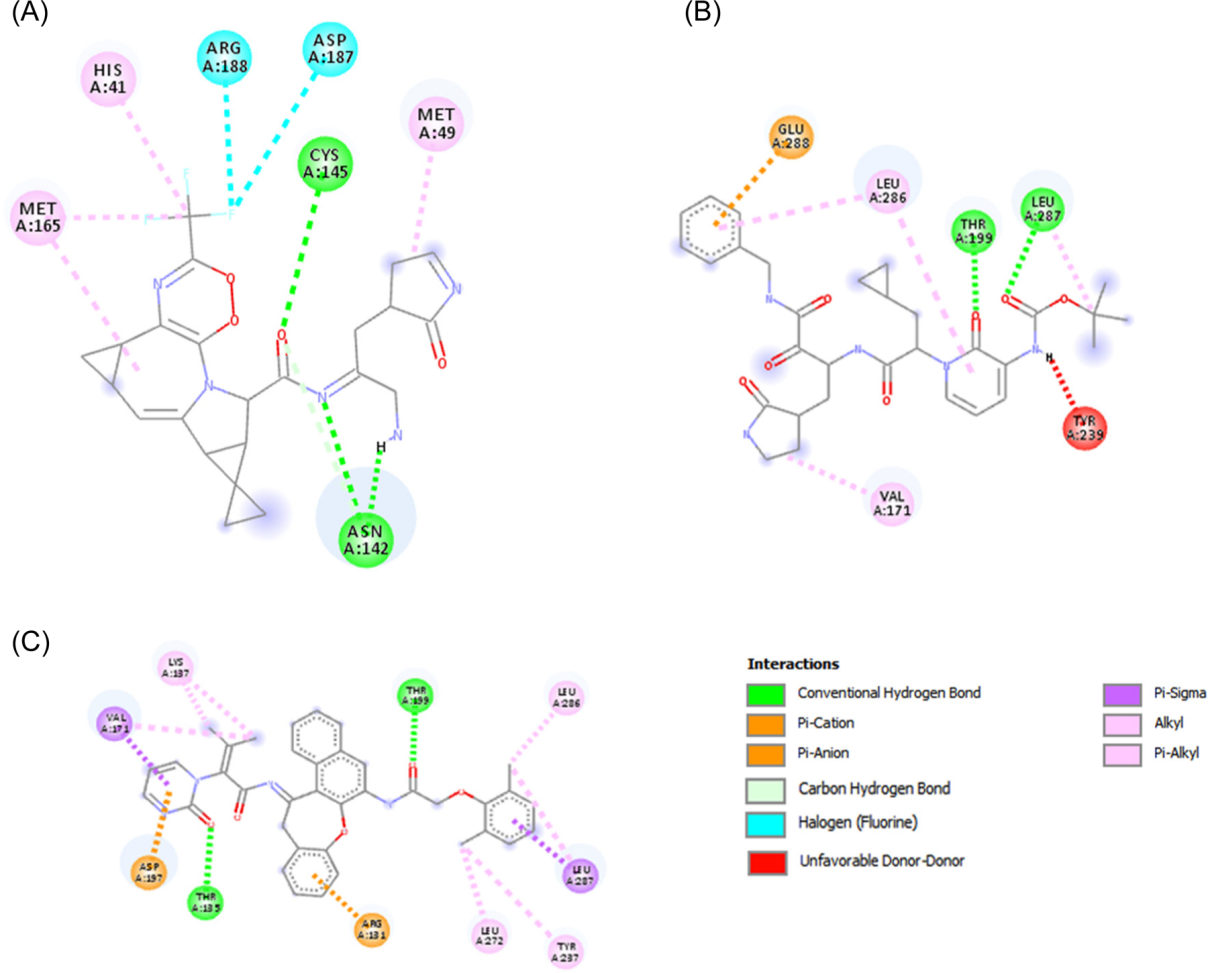
2D diagram of drug–protein interactions. Here, (A) interaction between Nirmatrelvir and main protease, (B) interaction between 13b and main protease, (C) interaction between Lopinavir and main protease.

**Figure 7:**
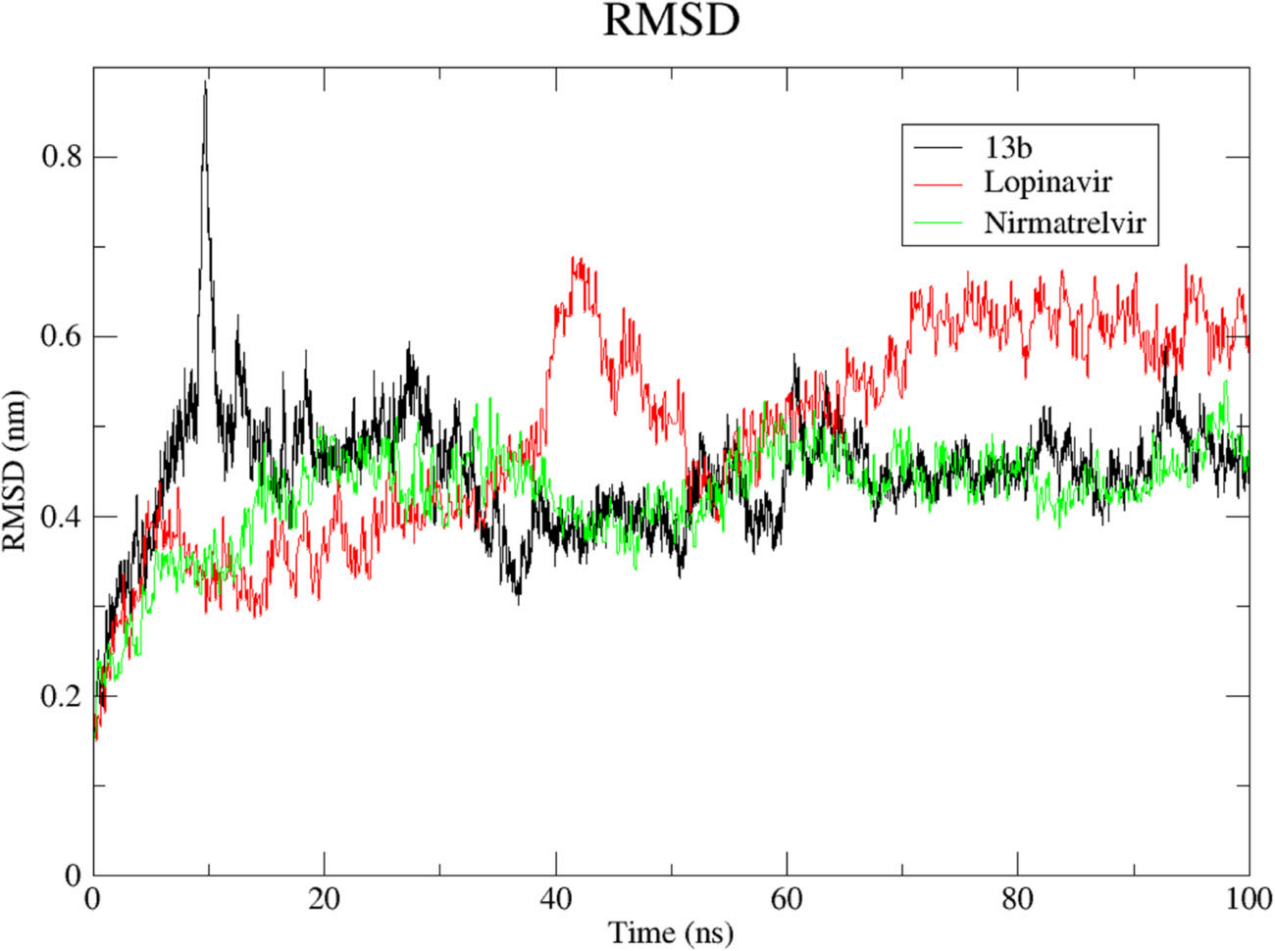
Root mean square deviation (RMSD) of the drug‐main protease complexes. After reaching the equilibrium state, the average RMSD for 13b‐main protease and Nirmatrelvir‐main protease was ~0.4 Å while ~0.6 Å for Lopinavir. Here, *x* axis represents the time interval of the simulation in ns while *y* axis for the RMSD value in nm.

Nirmatrelvir, Ritonavir, Ivermectin, Lopinavir, Boceprevir, 13b, N3, GC‐373, GC376, and PF‐00835231 were reported as effective against SARS‐CoV‐2, and most of them were currently in the clinical trials. To investigate the effectiveness of these drugs, we performed molecular docking against the main protease protein of Omicron. We found that mutations in the main protease of the Omicron variant did not significantly affect the binding energy for the interaction between these drugs and the main protease (Table 5). The binding affinity increased for Nirmatrelvir, 13b, and Lopinavir (Figure 5), despite no changes for Ivermectin, N3, and GC‐373. We found the lowest binding energy for Ivermectin against both wild and mutant main protease. The binding site for all drugs was similar, although the interacted amino acids were different (Figure 6, 7).

**Table 5:** Binding energy of promising drugs against main protease of omicron variant.

Drug name	Binding energy (kcal/mol)	
	Omicron	Wild
Ivermectin	−11.8	−11.8
Lopinavir	−9.6	−9.5
MPro 13b	−8.4	−8.1
Boceprevir	−8.4	−9.6
Ritonavir	−8.3	−8.5
GC‐373	−7.9	−7.9
Nirmatrelvir (Paxlovid)	−7.8	−7.7
GC‐376	−7.8	−8.8
PF‐00835231	−7.5	−7.8
MPro N3	−7	−7

### 3.7 Molecular dynamics simulation

**Figure 8:**
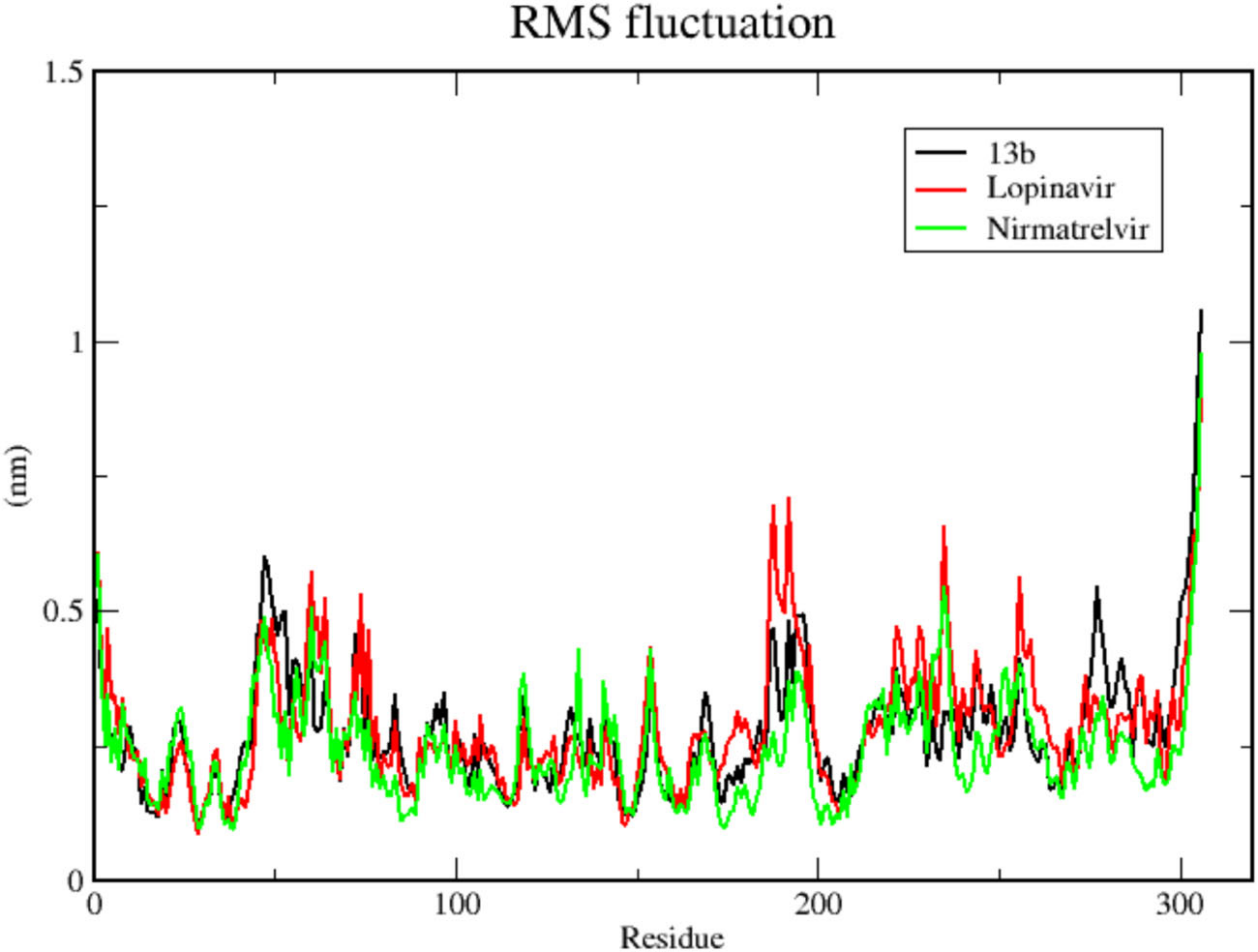
Root mean square fluctuation (RMSF) of the residues of main protease in complex with 13b, Lopinavir, and Nirmatrelvir. Here, *x* axis represents the number of residues of the receptor while *y* axis for RMSF value in nm. The average RMSF value for all three complexes was less than 0.5 nm while the maximum value for any residue was less than 1 nm.

**Figure 9:**
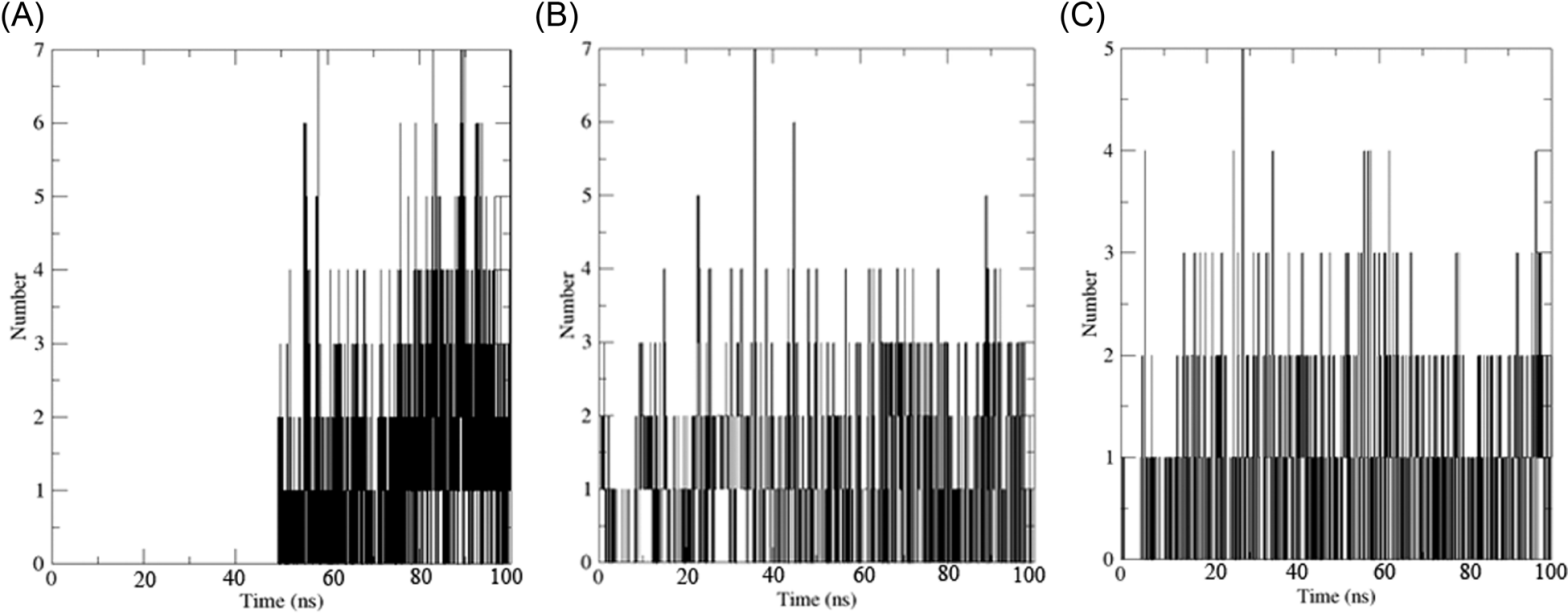
The number of H‐bonds involved in the interaction between the drugs and main protease. Here, (A) H‐bonds involved in 13b‐main protease, (B) H‐bonds involved in Lopinavir‐main protease, and (C) H‐bonds involved in Nirmatrelvir‐main protease.

We carried out MDS for the top three drugs including Nirmatrelvir, 13b, and Lopinavir, which showed an increase in effectiveness. In this simulation, the BFE of RMSD, RMSF, H‐bonds, and MM‐PBSA were calculated within the 100 ns of the time interval. Analysis of RMSD showed that the receptor was gone in an equilibrium state at around 40 ns, and the RMSD value was ~0.4 nm for Nirmatrelvir and 13b (Figure 6, 7). In the case of Lopinavir, the receptor was in an equilibrium state at around 70 ns and the RSMD value was slightly higher (~0.6 nm), compared to the other two (Figure 6, 7). The RMSF was mostly similar for all three drugs, and the average RMSF value was less than ~0.5 nm and the maximum RMSF for any residue was less than 1 nm (Figure 8). We also computed the number of H‐bond interaction between the drugs and the receptor during the simulation time. Lopinavir and Nirmatrelvir showed an average of three H‐bonds during the whole simulation time with a maximum number of seven and five H‐bonds, respectively (Figure 9). Surprisingly, in the case of 13b, the number of H‐bonds was not observed till 50 ns, but, after that, an average of four H‐bonds was seen with a maximum number of seven H‐bonds till the simulation ends (Figure 9). We also estimated the BFE using the MM‐PBSA method. The result was consistent with the docking results, while the lowest BFE was found for Lopinavir (−13.88 kcal/mol), followed by 13b and Nirmatrelvir (Table 6). van der Waals energy (∆*E*_vdw_) was contributed much for all three drugs to interact with the main protease of Omicron.

**Table 6:** MM‐PBSA binding free energies (in kcal/mol) of drugs in complex with main protease of Omicron.

Drugs	∆*E*_elec_	∆*E*_vdw_	∆*G*_bind_
Lopinavir	−20.38	−50.44	−13.88
13b	−16.21	−34.31	−10.23
Nirmatrelvir	−17.02	−39.39	−9.11

Abbreviations: ∆*E*_elec_, electrostatic energy; ∆*E*_vdw_, van der Waals energy; ∆*G*_bind_, the total binding free energy.

## 4 DISCUSSION

Twenty‐three months following the first emerging cases of COVID‐19 alongside its several variant classifications, another VOC, known as Omicron or B.1.1.529, was reported on November 26, 2021. While the world attempts to overcome the repercussions of COVID‐19, the high transmissibility and pathogenesis of the variants act as reversals. The conventional nature of RNA viruses to cause mutations within their genome raised the concerns associated with transmission and infection degrees. The following example would include the Delta variant, which has claimed millions of lives all around the world.^45^ On the other hand, the recently emerged the Omicron variant is the fifth VOC after Alpha, Beta, Gamma, and Delta. However, while the variants emerged through mutations, the mutational profile of Omicron is significantly different in comparison to the other variants; even though some genomic alterations resemble those of Beta and Delta, it is not exactly similar at the molecular level.^46^ The spike protein or S protein of Omicron is known to be the major site of mutation, which is labeled to have increased infectivity and transmissibility owing to its protein‐specific mutations. A similar mutation trend followed by previous VOCs, alongside other changes within the viral genome, also raises concerns associated with antiviral drug effectiveness, antibody therapies, and vaccine‐conferred immunity.^47^, ^48^ These rising concerns have reportedly rendered vaccines and antibody‐based therapies less effective, which have been proving to be the conventional lifesavers. Consequently, the need for novel effective antivirals and an evaluation of their targeted action against the virus remains of crucial significance. Through comprehensive evaluations, our study identified the structure‐based indifferences of the now emerging and dominant variant, Omicron. Further, this study elucidated not only the associated interactions between the RBD of spike protein and human receptors but also the effectiveness of the existing antiviral drugs.

Our extensive evaluations into the phylogenetic tree analysis initially indicated that the South Africa B.1.1.529 variant (i.e., Omicron) was quite similar to the SARS‐CoV‐2 B.1.620 variant of Germany. Surprisingly, B.1.620 was prevalent in Africa before emerging the Omicron variant which may support to conclude B.1.620 as the origin of the Omicron variant.^49^ Additionally, the B.1.620 variant had D614G mutation, which was shown responsible for an increased SARS‐CoV‐2 infection pattern.^50^ Therefore, it can be credited that the Omicron variant follows a similar infectivity trend owing to its phylogenetic similarity that contributes to the current surging COVID‐19 cases worldwide. Besides, the South Africa B.1.1.529 Omicron variant was found to have an overall of 50 nonsynonymous mutations—a majority of which were found in the spike protein. Moreover, the identification‐based analysis revealed that the Omicron variant consisted of mutations in polyprotein ab, spike protein, envelope protein, membrane glycoprotein, and nucleocapsid phosphoprotein. And polyprotein ab is cleaved into several nonstructural proteins. We also found mutations in these proteins, including papain‐like protease nsp3, nsp4, 3CL protease nsp5, nsp6, RNA‐dependent RNA polymerase nsp12, and proofreading exoribonuclease nsp14. Seemingly, a study by Zhu et al.^51^ observed through *Drosophila* viability assays that nsp6 was one of the most pathogenic SARS‐CoV‐2 genes, capable of triggering lethal consequences individually and, at the same time, was labeled as one of the primary determinants of COVID‐19 pathogenesis.^51^ Mutations in this protein could affect the intracellular survival of the virus and could also make a significant modification in viral pathogenicity.^52^ Three consecutive deletions and a substitution mutation in the genome sequence of Omicron possibly indicate a reduced pathogenicity. Mutations of nsp3, a major protein for the SARS‐CoV‐2 replication, suggest a lower replication rate and infectivity. These two proteins, along with nsp4 and nsp5, are known for double‐membraned vesicle inductions and localizations of cleaved maps.^53^, ^54^ While nsp12 and nsp14, which are required to mediate polymerase and exonuclease activities, had been shown genetic alterations presumably affecting their viral load, other structural proteins were also mutated. These include the Spike (S) protein which is responsible for facilitating the membrane fusion and viral entry^55^; the Envelope (E) protein which is contributory to virus morphogenesis and pathogenesis^56^; the Membrane (M) protein which aids membrane fusion through its initial attachment to the S protein and surface receptors of the host^57^, ^58^; the Nucleocapsid (N) protein moderates replication and viral RNA synthesis, transcription and metabolism associated with infected cells and additionally provides stability to the RNA inside the cell.^59^, ^60^, ^61^ While the roles of these generalized protein and their mutations may help hypothesize Omicron as less pathogenic than others, only further research into their gene‐specific mutations of the Omicron variant may act as better pointers for characteristic identification.

With a maximum of mutations in the spike (S) protein, the mutant RBD of that very protein was modeled and validated for further analysis. We studied the chosen human receptors, including ACE2, NRP1, DPP4, and BSG, through molecular docking processes to understand their interactions with the mutant RBD of the spike protein. Notably, the results indicated a decreased binding energy for interacting mutant RBD with ACE2 and a significantly increased binding energy for interacting mutant RBD with NRP1, compared to the wild‐type RBD. However, both DPP4 and BSG showed higher binding energies in either form, compared to the former two human receptors. Spike protein RBD of the Omicron variant contains 11 mutations, and they would be the responsible elements for increasing binding affinity for the interaction with ACE2. Mutations in RBD occurred for an optimization of the binding affinity because it would be advantageous for the virus to enhance its transmissibility. Barton et al.^62^ reported that three mutations of RBD: N501Y, E484K, and S477N enhance the binding affinity for interaction with ACE2. Surprisingly, all these mutations were available in the spike protein RBD of the Omicron variant, implying for an increase in their binding affinity. Acknowledging the characteristic label of infection 70 times faster than the deadly Delta variant and the initial COVID‐19 strain being less severe,^63^ a generalized hypothesis could be provided alongside the results of our study. Owing to its greater infectivity but lower pathogenicity, as a comparison to its receptor‐binding capacity, it is hypothesized that ACE2 is responsible for increased infectivity whereas NRP1 is associated with increased pathogenicity; in cases of the Omicron variant, the increased binding affinity for ACE2 corresponds to its greater infection rate, while the decreased binding affinity for NRP1 corresponds to a decreased pathogenicity.

Furthermore, we also conducted analyses based on the drug effectivity for Nirmatrelvir, Ritonavir, Ivermectin, Lopinavir, Boceprevir, 13b, N3, GC‐373, GC376, and PF‐00835231. The evaluation of these drugs to determine their interaction with their targeted main protease of the Omicron variant revealed that mutations within the major interacting protein did not hamper the binding energy at all, except for Boceprevir and GC‐376, which showed increased binding energy. The increased binding affinity of Nirmatrelvir (Paxlovid), 13b, and Lopinavir may indicate their greater drug efficacy against this Omicron variant compared to previous variants. This result was also validated by MDS. The lower RMSD and constant number of H‐bonds during the whole simulation indicated the strong interaction between the drugs and the main protease. The lower RMSF also indicated the strong interaction for all three drugs against the receptor.^64^ Additionally, we found that the BFE estimated by the MM‐PBSA method was consistent with the docking result and van der Waals energy, which played a crucial role in making the strong interaction for all three drugs against the receptor. Recently, two independent studies experimentally proved the activity of Nirmatrelvir against Omicron.^65^, ^66^ From our study, Ivermectin showed the highest binding affinity, suggesting to be the most effective drug candidate against the Omicron variant. While these hypotheses hold great value and may provide significant insights into the therapeutic strategies, further research is crucial to authenticate these statements.

## 5 CONCLUSION

The world is now afraid of the highly infectious Omicron variant, and research is required to know about this variant. Our study gave an insight into its probable molecular consequences about infectivity and pathogenicity of the Omicron variant. The study also demonstrated that the highly infectious B.1.620 strain would be the origin of the Omicron variant, and mutations in all major proteins made Omicron less pathogenic. Through docking analysis, we revealed that the mutations in spike protein increased its binding affinity for its main receptor ACE2 while decreased binding affinity for its coreceptor NRP‐1. All the promising drugs that target the main protease would also be effective against this variant; however, Ivermectin shows the strongest binding affinity, and Nirmatrelvir (Paxlovid), 13b, and Lopinavir may be more effective against this variant.

## AUTHOR CONTRIBUTIONS

**Md Sorwer Alam Parvez**: Conceptualization; methodology; formal analysis; data interpretation; validation; visualization; original draft preparation. **Manash Kumar Saha**: Methodology; software; visualization. **Md Ibrahim**: Formal analysis. **Yusha Araf**: Formal analysis; original draft preparation and editing. **Md Taufiqul Islam:** Validation. **Gen Ohtsuki**: Supervision, writing—review & editing. **Mohammad Jakir Hosen**: Supervision, writing—review & editing.

## CONFLICTS OF INTEREST

The authors declare no conflicts of interest.

## ETHICS STATEMENT

This study did not deal with human subjects and biological materials. All open‐source data were analyzed, in which all personal information was anonymized, and no data allowing individual identification was retained. Therefore, no ethics approval and no informed consent were required.
